# Sociodemographic Drivers of Recruitment and Attrition in Digital Neurological Research: Longitudinal Cohort Study

**DOI:** 10.2196/83432

**Published:** 2026-02-25

**Authors:** Peyman Nejat, Ashley D Bachman, Vicki M Stubbs, Joseph R Duffy, John L Stricker, Vitaly Herasevich, David T Jones, Rene L Utianski, Hugo Botha

**Affiliations:** 1 Department of Anesthesiology and Perioperative Medicine, Mayo Clinic, Rochester, MN, United States; 2 Office of Digital Innovation, Center for Clinical And Translational Science, Mayo Clinic, Rochester, MN, United States; 3 Department of Neurology, Mayo Clinic, 200 First St SW, Rochester, MN, 55905, United States, 1 5072842511; 4 Department of Information Technologies, Mayo Clinic, Rochester, MN, United States

**Keywords:** digital recruitment, neurological research, participation disparities, sociodemographic factors, digital divide

## Abstract

**Background:**

Digital recruitment methods offer opportunities to address challenges in clinical research participation, particularly in neurology. However, the impact of digital approaches across socioeconomic and demographic groups remains inadequately understood.

**Objective:**

This study investigates the influence of sociodemographic factors on recruitment and attrition in a remote neurological research cohort, mapping participation pathways and identifying disparities to inform inclusive digital strategies.

**Methods:**

We conducted a nonexperimental, observational longitudinal cohort study at Mayo Clinic using patient-portal invitations between March and July 2024 as part of a remote speech capture study. Eligibility criteria included age 18 years and older, US residence, and English proficiency. Of 5846 invited patients, progression was tracked across checkpoints (invitation, eligibility screening, electronic consent, and task completion) using Epic (Epic Systems Corporation) to obtain demographic information, Qualtrics (Qualtrics, LLC) for screening, PTrax (a Mayo Clinic–developed Participant Tracking System) for consent tracking, and the recording platform. Socioeconomic context was assessed using the Housing-based Socioeconomic Status (HOUSES) index, where higher values indicate higher socioeconomic status, and the Area Deprivation Index (ADI), where higher values reflect greater neighborhood disadvantage. Data diagnostics included Anderson-Darling tests for non-normality and Little missing completely at random (MCAR) test to characterize missingness. Associations between participation outcomes and age, sex, urbanicity, and socioeconomic indices were examined using nonparametric tests. Exact *P* values and 95% CIs are reported. Analyses were conducted using BlueSky Statistics (BlueSky Statistics, LLC) and the Python *SciPy* package.

**Results:**

Overall, 415 out of 5846 participants (7.1%) completed all study requirements. Completers were older (median age 66.4, IQR 56.0-72.5; 95% CI 65.1‐67.6 years) than noncompleters (median age 62.8, IQR 47.5-72.7; 95% CI 62.2‐63.2 years; *P*<.001). Participants from more socioeconomically disadvantaged neighborhoods were less likely to respond (invitation nonresponder median ADI 45.0, IQR 29.0-63.0 vs interested median ADI 42.0, IQR 27.0-59.0; *P*<.001), and completers had slightly lower ADI ranks than noncompleters (median 41.0, IQR 27.0-56.0 vs median 44.5, IQR 28.0-62.0; *P*=.04). Urban participants enrolled faster (median 32.0, IQR 9.0-58.0; 95% CI 31.0‐37.0 days) than rural (median 41.0, IQR 22.0-65.0; 95% CI 37.0‐49.0 days; *P*=.01). Female participants responded slower (median 38.5, IQR 14.8-66.3; 95% CI 35.0‐41.0 days) than males (median 32.0, IQR 8.0-57.5; 95% CI 29.0‐38.0 days; *P*=.01). No significant differences were observed for the HOUSES index, and device type was unrelated to completion or timelines. Missingness for key variables was completely at random (MCAR *χ*²_3_=3.45; *P*=.24).

**Conclusions:**

Digital recruitment does not overcome traditional barriers to participation and may introduce new disparities related to age, urbanicity, and neighborhood disadvantage. These findings inform inclusive digital research strategies, including multichannel outreach, age-specific engagement, and rural technical support. This study applies longitudinal pathway analysis to digital neurology recruitment, offering actionable insights for improving inclusivity in remote research.

## Introduction

Participant recruitment is a critical component of medical research studies, and the rate of participation varies significantly depending on the type of study, the setting, and the population involved. As an example, recent data suggest that enrollment rate in cancer trials is around 6.3% to 7.1% [1,2]. Enrollment rates may also differ among different populations; specifically, they can be lower among minority, pediatric, and geriatric populations [3,4]. Poor recruitment often results in underpowered studies, which lack the necessary sample size to detect meaningful differences between distinct groups. This can lead to statistically nonsignificant results even when there are clinically relevant effects [5]. Despite these efforts and the growing adoption of digital recruitment tools, recent analyses confirm that these challenges persist with participation patterns continuing to vary by age, sex, and socioeconomic status (SES) [6]. This underscores a critical point that digital methods may not fully mitigate traditional barriers and could introduce new inequities that influence who participates in research.

Digital tools, including social media platforms, mobile apps, electronic health records, patient portals, and electronic consent, have expanded the reach of research recruitment efforts by enabling targeted outreach to specific demographics and geographic areas [7-10]. These approaches can engage populations previously hard to reach through traditional methods, but evaluations report inconsistent effects on diversity and efficiency across settings [11]. A recent systematic review cataloged the spectrum of digital technologies deployed for recruitment and highlighted the still-limited evidence that any one approach reliably improves inclusion of underrepresented groups [6].

A central challenge for digital recruitment is the digital divide, defined as persistent inequities in broadband access, device availability, and digital literacy that map closely onto socioeconomic, geographic, and age-related lines [12]. These inequities can depress response rates and create systematic attrition in specific subgroups even when outreach is delivered digitally. Recent studies and reviews caution that digitalization can reproduce or widen participation gaps if considerations are not built into recruitment strategies [13]. Within health care systems, portal-based recruitment shows promise but exhibits differential response patterns across patient characteristics, underscoring the need to track impacts on disparity as programs scale. Moreover, scoping reviews in specific domains suggest that mixed, multichannel recruitment strategies (digital and offline) may improve inclusivity compared with relying on a single modality [11]. These disparities are often more pronounced among disadvantaged groups, including racial and ethnic minorities and older adults, who may have limited access to the internet and lower digital literacy levels [14-16]. For instance, studies have shown that older adults and African American patients are less likely to use digital health portals compared to their younger and White counterparts, highlighting a significant gap in technology use [16]. Here, the term “White” reflects the racial classification used in the original study, which reported race and ethnicity separately and did not provide ethnicity‑specific breakdowns. Similarly, individuals from lower SES neighborhoods often have reduced access to the internet and lower health literacy, which can hinder their ability to engage with digital health technologies effectively [15]. While digital tools have the potential to improve the representativeness of trial participants, there is limited evidence supporting their effectiveness in recruiting underrepresented groups [8,17]. This underscores the need for targeted interventions and strategies to bridge the digital divide and ensure equitable access to digital health resources [18,19].

Digital recruitment has become especially popular with the increased interest in artificial intelligence (AI) in health care, which requires large and representative datasets to train. For example, the Bridge2AI-Voice program has the explicit goal to create “an ethically sourced flagship dataset to enable future research in artificial intelligence” [20]. Such datasets are typically assembled through digital recruitment workflows, underscoring the need to understand how these methods shape participant diversity. Our group has similarly endeavored to obtain a large bank of speech recordings focused on neurological disorders, with the goal of subsequently using these data to train AI models, through a primarily digital recruitment approach. Given concerns about the digital divide and its potential impact on representativeness, the broader speech capture study offered the ideal setting to formally investigate these issues in depth. This study aims to characterize recruitment and attrition patterns in a remote neurology cohort, quantify associations with sociodemographic factors (age, sex, neighborhood deprivation, housing-based SES, and urbanicity), and identify drop-off points to inform strategies for digital recruitment for all participants.

## Methods

### Study Design and Setting

This analysis was nonexperimental and observational, using a longitudinal cohort design to evaluate sociodemographic factors influencing recruitment and attrition in a remote speech capture study. This study was conducted in accordance with the American Psychological Association (APA) Journal Article Reporting Standards (JARS; American Psychological Association, 2018; refer to Multimedia Appendix 1 for the completed JARS-Quant checklist) [21]. The overarching speech capture study aims to remotely collect speech samples from patients with neurologic diseases to develop an easy-to-use and cost-effective screening tool for predicting disease progression. The parent speech capture study extends beyond the March-July 2024 analysis window; for this report, we used data collected within that period. The research was conducted at Mayo Clinic.

### Inclusion and Exclusion Criteria

Individuals were eligible if they met the following criteria: (1) adults aged 18 years or older, (2) residing in the United States, and (3) able to communicate in English via spoken language.

### Data Collection and Participant Characteristics

Patient identification was conducted using the Mayo Clinic Electronic Health Record (Epic, developed by Epic Systems Corporation), and invitations to complete an eligibility survey were sent via the patient portal using Qualtrics (developed by Qualtrics, LLC) [22]. The eligibility survey also assessed participants’ understanding of the study and asked whether they had a legally authorized representative responsible for financial or health care decisions. Once interest and eligibility were confirmed, participants received a PDF of the consent form to sign electronically via AdobeSign (Adobe Inc) through Mayo Clinic–developed Participant Tracking System (PTrax). PTrax is an institutional research software program designed to streamline informed consent processes, manage participant status, track enrollments and accruals, and provide reporting and analytics. Recruitment followed a convenience sampling approach, targeting patients with upcoming neurology appointments accessible via the institutional patient portal. After consent was obtained, participants were sent a secure message in the patient portal with a link to the speech recording platform and instructions.

We exported longitudinal record data for all eligible patients invited to the speech capture study between March and July 2024. This resulted in a total sample of 5846 participants, reflecting all patients meeting the inclusion criteria during the recruitment window. Race was extracted from the electronic health record, where “White” reflects a race category and is distinct from ethnicity. Ethnicity (eg, Hispanic or Latinx origin) was not included in the dataset for this secondary analysis; therefore, individuals categorized as “White” may include participants of diverse ethnic backgrounds. The invited cohort had a median (IQR) age of 63 (48-72) years, with 56.2% (3283/5846) female and 93.7% (5478/5846) identifying as White. Urbanicity distribution was 56.5% (3303/5846) urban, 23.3% (1363/5846) rural, and 20.2% (1180/5846) urban cluster. This secondary analysis included all eligible patients invited during the recruitment window of the overarching speech capture study. No a priori power calculation was performed because the analysis was observational and descriptive. Precision was conveyed using 95% CIs for medians and IQRs. Data were drawn from Epic (demographics), Qualtrics (survey responses), PTrax (consent tracking), and the speech recording platform (task completion).

### Measures and Covariates

SES was assessed using the Housing-based Socioeconomic Status (HOUSES) index and the Area Deprivation Index (ADI) national rank. The HOUSES index is a practical and adaptable tool for assessing SES using housing data. It effectively correlates with traditional SES measures and predicts various health outcomes [23]. Higher HOUSES index values indicate higher SES, while lower values indicate lower SES. The ADI national rank measures neighborhood socioeconomic disadvantage based on factors such as income, education, employment, and housing characteristics [24,25]. Higher scores indicate greater disadvantage. ADI is widely used in public health research to assess the impact of socioeconomic context on health outcomes.

Primary outcomes included study completion and time to enrollment. Exposures and predictors included age, sex, urbanicity (urban, rural, or urban cluster), ADI national rank, and the HOUSES index. Potential confounders considered were device type and urbanicity. Urbanicity was treated as an exposure in primary analyses and as a potential confounder in models examining associations between socioeconomic indices and participation outcomes. The collected data included participants’ age, sex assigned at birth (hereafter referred to as sex), date of invitation, recruitment process checkpoints and dates, and devices used for speech recording. Residence information was used to measure the HOUSES and ADI index and to classify participants by urbanicity (urban, rural, or urban cluster).

### Quality of Measurements

This analysis relied exclusively on data exported from established operational systems (Epic for demographics, Qualtrics for eligibility responses, PTrax for participant status tracking, and the recording platform for task completion). No new training, instrumentation, or measurement procedures were implemented specifically for this analysis. All timestamps and statuses were generated by the source systems as part of routine workflows. Data were deidentified before analysis.

### Analytic Strategy

**Figure 1.**
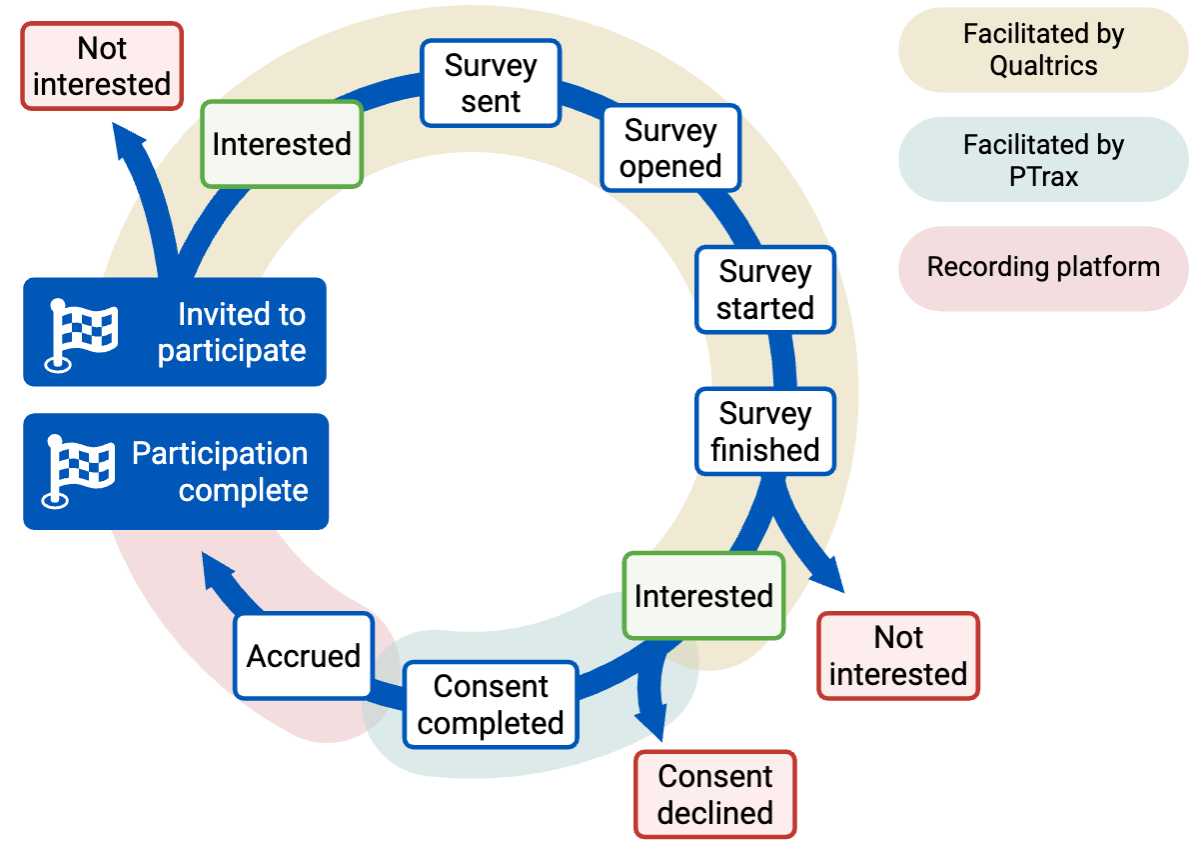
Simplified view of the participation enrollment pathways in a longitudinal digital neurology research study. PTrax: Participant Tracking System.

The longitudinal time series data for each patient undergoing the participation process were standardized to align with specific checkpoints, as demonstrated in Figure 1. At each step, it was possible for patients not to respond to the research coordination team, which was defined as the “No Response” stage. After providing consent, participants were also free to withdraw it at any time. While most individuals followed one of the typical pathways depicted in Figure 1, there were 83 cases (1.4% of the total 5846 cases) in which participants deviated from these paths, necessitating intervention by research coordinators. These atypical cases were primarily attributed to personal circumstances or the involvement of a legally authorized representative.

Kruskal-Wallis and Wilcoxon rank-sum tests were used to compare the median age, socioeconomic indices, and time taken to reach different steps of the study. These nonparametric tests were selected due to non-normal distribution of key variables, as confirmed by the Anderson-Darling test (*P*<.001). They are appropriate for comparing medians across groups and handling skewed data. Analysis was conducted at 2 levels: at each checkpoint and through an end-to-end investigation of participants who completed the study. For each path, a participant could take at each checkpoint, age, ADI national rank, and HOUSES index were compared to identify statistically significant differences, using a 2‑sided alpha level of .05. At the end-to-end level, additional comparisons were made across sex, urbanicity (urban, rural, and urban cluster), ADI national rank, and HOUSES index to evaluate differences in time taken to complete participation, from initial invitation to accrual, and whether participants completed the study, regardless of the path taken. Analyses were conducted using BlueSky Statistics version 10.3.4 (developed by BlueSky Statistics, LLC) and Python *SciPy* package version 1.16. Exact *P* values are reported, with *P*<.001 used where appropriate. No effect sizes were calculated for analyses.

### Data Diagnostics

Since both Kruskal-Wallis and Wilcoxon rank-sum tests require at least 5 data points in each comparison category, pathways with fewer than 5 participants without missing values were excluded from the analysis. This threshold was applied to ensure statistical validity and avoid unreliable comparisons in small subgroups, as no randomized assignments or masking strategies were implemented. To further assess the nature of missing data across key sociodemographic variables, including age, ADI national rank, and HOUSES index, we conducted Little's missing completely at random (MCAR) test [26]. This test evaluates whether data are MCAR, which informs the appropriate handling strategy. Based on the results, we adopted a pairwise deletion approach for statistical analyses, allowing each test to include all available cases for the specific variable of interest. This method was chosen to preserve sample size and maintain statistical power, particularly in subgroups with limited data. This method was chosen to preserve sample size and maintain statistical power, particularly in subgroups with limited data. No outlier removal or variable transformations were applied.

### Ethical Considerations

The overarching speech capture study was reviewed and approved by the Mayo Clinic Institutional Review Board (#22-002430). Informed consent was obtained electronically for the primary speech capture study via Adobe Sign. For this secondary analysis of recruitment and attrition patterns, which used deidentified data from the primary study, the institutional review board determined that additional approval was not required. All data were deidentified prior to analysis to ensure participant confidentiality. No compensation was provided for participation. No identifiable images of participants are included in the manuscript or any supplementary materials.

## Results

### Overview of the Invited Cohort

A total of 5846 patients were invited to participate in the study between March and July 2024. Of these participants, 3283 (56.2%) were female, 2560 (43.8%) were male, and 3 (0.1%) were unknown. Most participants (5478/5846, 93.7%) identified as White. The age distribution ranged from 18 to 96 years, with a median (IQR) age of 63 (48-72) years (N=5846; 95% CI 62‐63). The narrow CI suggests high precision in estimating the median age of the invited cohort. Regarding urbanicity, 56.5% (3303/5846) of invited participants resided in urban areas, 23.3% (1363/5846) in rural areas, and 20.2% (1180/5846) in urban clusters (Table 1). Following accrual completion, participants used various devices to access the recording platform. Apple-based mobile devices (iPhone and iPad) were most frequently used (141/415, 34.0%), followed by Windows-based computers (134/415, 32.3%), Apple-based computers (82/415, 19.8%), Android-based mobile devices (52/415, 12.5%), and other devices (6/415, 1.4%).

**Table 1.** Demographic characteristics of 5846 patients invited to participate in a remote speech capture study for neurological research at Mayo Clinic between March and July 2024.

Characteristic	Participants, n (%)
Sex	
Female	3283 (56.2)
Male	2560 (43.8)
Unknown	3 (0.1)
Race	
White	5478 (93.7)
Black or African American	97 (1.7)
Choose not to disclose	65 (1.1)
Other	206 (3.5)
Age (years)	
18‐33	569 (9.7)
34‐49	999 (17.1)
50‐64	1607 (27.5)
65‐80	2203 (37.7)
≥81	468 (8.0)
Population	
Urban area	3303 (56.5)
Rural area	1363 (23.3)
Urban cluster	1180 (20.2)
Device used for participation	
Apple-based mobile device	141 (34.0)
Windows-based computer	134 (32.3)
Apple-based computer	82 (19.8)
Android-based mobile device	52 (12.5)
Other devices	6 (1.4)

**Figure 2.**
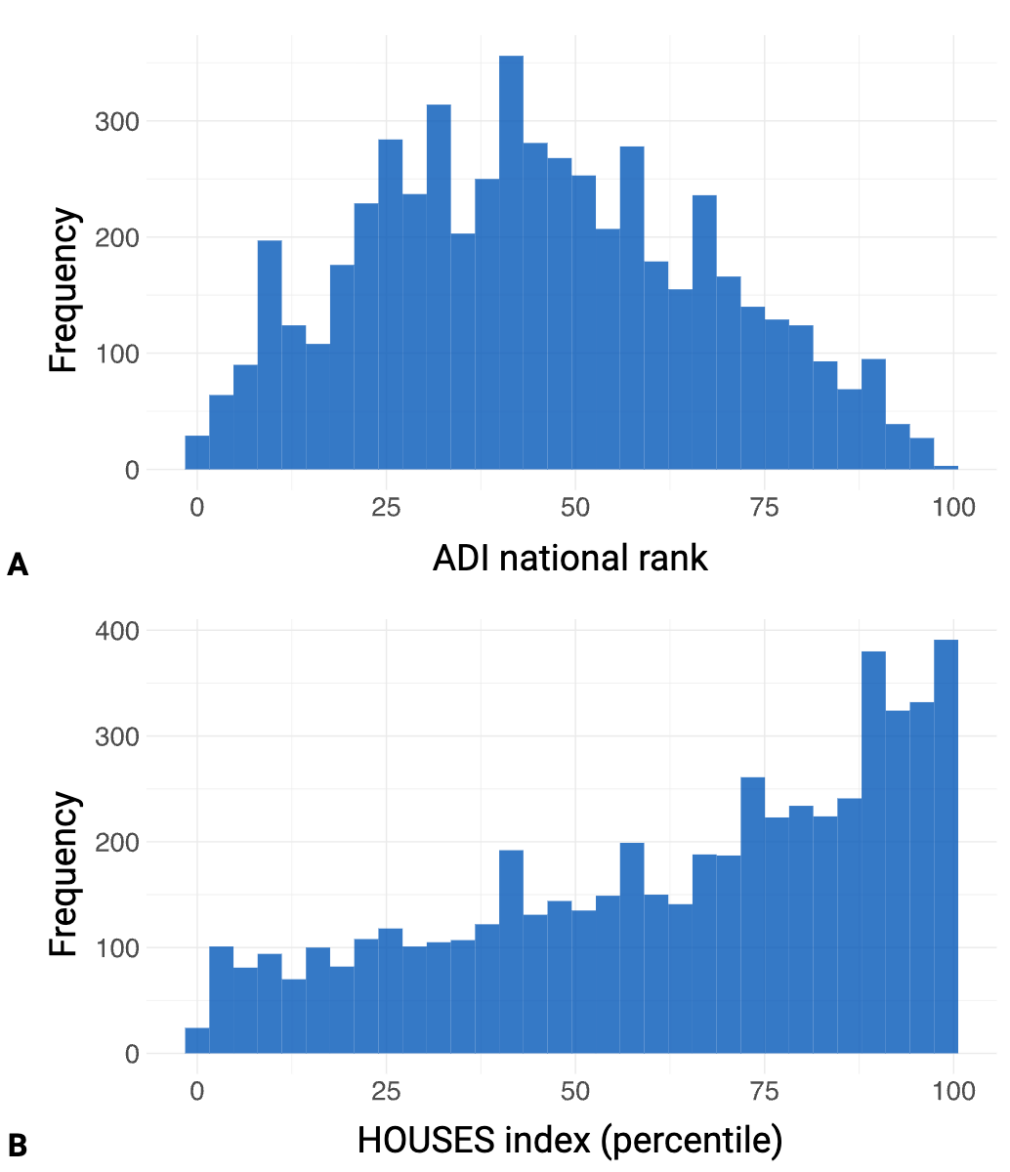
Distribution of socioeconomic measures among participants in a remote speech capture study for neurological research. ADI: Area Deprivation Index. HOUSES: Housing-based Socioeconomic Status.

The ADI national rank of participants spanned the entire range from 1 to 100, with a median (IQR) of 44 (28-61; n=5403; 95% CI 43‐45), indicating representation across diverse socioeconomic backgrounds. Similarly, the HOUSES index percentile ranged from 1 to 100, with a median (IQR) of 70 (43-88; n=5439; 95% CI 69‐71), demonstrating considerable variability in housing conditions among participants. The tight CI reflects reliable estimation despite the wide IQR, which indicates heterogeneity in housing-based SES. Neither age, ADI national rank, nor HOUSES index exhibited normal distribution according to the Anderson-Darling test (*P*<.001 for all variables), confirming the appropriateness of nonparametric statistical methods used in subsequent analyses. Figure 2 illustrates the distribution of the HOUSES index percentile and ADI national rank, with panel A showing ADI national ranks and panel B showing HOUSES index percentiles.

To assess the nature of missing data, Little MCAR test was conducted on age, ADI national rank, and HOUSES index and yielded *χ*²_3_=3.45; *P*=.24. This nonsignificant result indicates that missingness was not systematically related to other variables, supporting the assumption that data were MCAR. Accordingly, pairwise deletion was applied in subsequent analyses, a strategy chosen to preserve sample size and maintain statistical power while ensuring valid comparisons between study completers and noncompleters.

### Comprehensive Participation Analysis

Significant differences were observed between participants who completed the study and those who did not across several demographic and socioeconomic factors. Age was higher among completers (median 66.4, IQR 56.0-72.5; 95% CI 65.1‐67.6 years) compared to noncompleters (median 62.8, IQR 47.5-72.7; 95% CI 62.2‐63.2 years; *P*<.001), suggesting that older individuals were more likely to participate (Table 2). The narrow CIs for both groups indicate high precision in estimating age differences, reinforcing the robustness of this finding. Participants who completed the study also resided in slightly less socioeconomically disadvantaged areas, as indicated by lower ADI national ranks (median 41.0, IQR 27.0-56.0; 95% CI 39.0‐43.0 vs median 44.5, IQR 28.0-62.0; 95% CI 43.0‐45.0; *P*=.04). Although the difference is statistically significant, the overlapping CIs suggest that the magnitude of this effect is modest and should be interpreted cautiously. No significant differences were found in HOUSES percentiles, indicating that individual HOUSES may exert less influence on participation compared to neighborhood-level disadvantage.

**Table 2.** Comparison of socioeconomic and demographic characteristics between participants who completed the remote speech capture and those who did not. The Wilcoxon rank-sum test was applied to assess differences between groups.

Variable and group	Number of participants	Median (IQR)	95% CI	*P* value
Age (years)				<.001
Participation not complete	5431	62.8 (47.5‐72.7)	62.2‐63.2	
Participation complete	415	66.4 (56.0‐72.5)	65.1‐67.6	
ADI^a^ national rank				.04
Participation not complete	5018	44.5 (28.0‐62.0)	43.0‐45.0	
Participation complete	385	41.0 (27.0‐56.0)	39.0‐43.0	
HOUSES^b^ percentile				.76
Participation not complete	5052	70.0 (42.0‐88.0)	68.0‐71.0	
Participation complete	387	71.0 (44.0‐88.0)	66.0‐75.0	

a ADI: Area Deprivation Index.

b HOUSES: Housing-based Socioeconomic Status.

Sex differences in enrollment time were significant, with female participants taking longer to enroll than males (median 38.5, IQR 14.8-66.3; 95% CI 35.0‐41.0 vs median 32.0, IQR 8.0-57.5; 95% CI 29.0‐38.0 days; *P*=.01; Table 3). The relatively narrow CIs for both groups indicate precise estimates of enrollment time differences, reinforcing the statistical significance of this finding. Additionally, urbanicity influenced the time to complete enrollment. Participants from urban areas enrolled more quickly than those from rural or urban cluster regions (median 32.0, IQR 9.0-58.0; 95% CI 31.0‐37.0 vs median 41.0, IQR 22.0-65.0; 95% CI 37.0‐49.0 days and median 40.0, IQR 13.0-71.0; 95% CI 33.0‐49.0, respectively; *P*=.01). The wider CIs for rural and urban cluster groups suggest greater variability in enrollment time compared to urban participants, possibly reflecting differences in access or engagement. However, no significant differences were found in completion time across sex or urbanicity. No significant associations were detected between HOUSES indices and study completion. Additionally, no significant relationship was found between the device type used for task completion and the completion or participation time. These null findings suggest that SES and device type did not influence completion dynamics in this cohort.

**Table 3.** Enrollment and completion times stratified by urbanicity and sex among participants in a remote speech capture study for neurological research. Statistical significance was assessed using the Wilcoxon rank-sum test or the Kruskal-Wallis test, depending on the category count.

Variable	Number of participants	Median (IQR)	95% CI	*P* value
Enrollment time (days)				.01
Rural	150	41.0 (22.0‐65.0)	37.0‐49.0	
Urban area	532	32.0 (9.0‐58.0)	31.0‐37.0	
Urban cluster	141	40.0 (13.0‐71.0)	33.0‐49.0	
Completion time (days)				.70
Rural	74	20.0 (12.3‐36.0)	15.5‐22.0	
Urban area	269	20.0 (10.0‐32.0)	17.0‐22.0	
Urban cluster	72	19.5 (7.0‐38.8)	12.5‐27.0	
Enrollment time (days)				.01
Male	343	32.0 (8.0‐57.5)	29.0‐38.0	
Female	480	38.5 (14.8‐66.3)	35.0‐41.0	
Completion time (days)				.95
Male	181	20.0 (10.0‐32.0)	16.0‐22.0	
Female	234	20.0 (9.0‐35.0)	15.5‐22.0	

### Step-by-Step Participation Analysis

The majority of invited participants either did not read or did not respond to the initial invitation via Epic (n=2736) or expressed no interest (n=1752). Among the 1358 participants who initially expressed interest, 415 (30.6%) ultimately completed the study in its entirety. Throughout various stages of the recruitment process, a total of 3346 participants failed to respond to follow-up communications from the research coordination team.

Analysis of participant age across different pathways revealed that individuals who did not respond to the invitation or eligibility check were significantly younger than those who proceeded toward study completion. This age disparity contributed to an increase in the median age of participants completing the study (66.4, IQR 56.0-72.5; 95% CI 65.1‐67.6) years compared to the overall invited cohort (62.8, IQR 47.5-72.7; 95% CI 62.2‐63.2) years. The narrow CIs for both estimates indicate high precision, reinforcing confidence in the observed age-related attrition pattern. A similar pattern was observed among the 95 participants who withdrew consent after initially providing it but before being accrued for the recording session, with these participants having a median age of 55.8 years compared to 66.3 years for those who continued with the recording. This suggests that younger participants were disproportionately represented among early dropouts.

**Figure 3.**
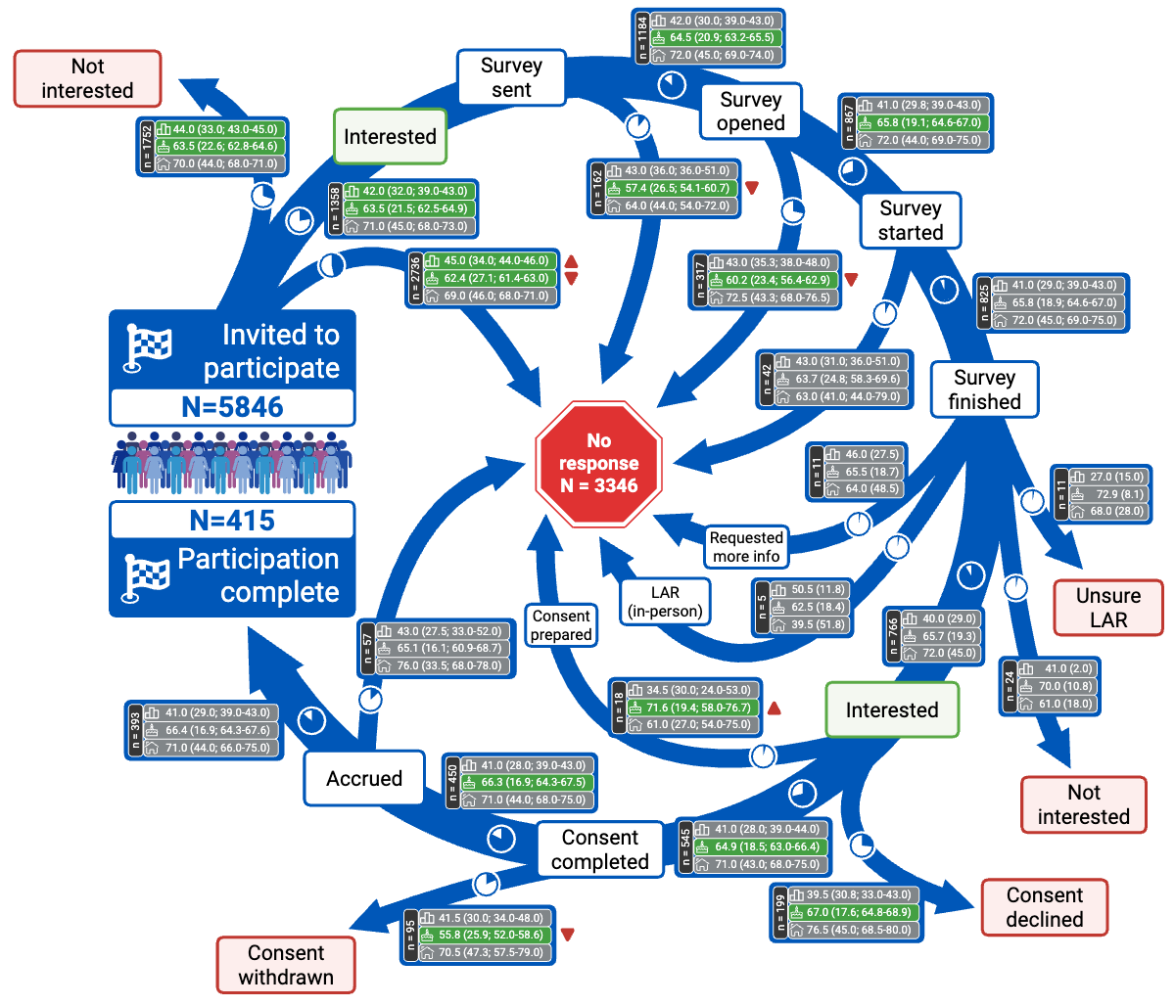
Step-by-step analysis of participant recruitment and attrition in a longitudinal digital neurology research study conducted at Mayo Clinic between March and July 2024. The figure illustrates participant flow across recruitment checkpoints, with descriptive statistics for Area Deprivation Index (ADI), age, and Housing-based Socioeconomic Status (HOUSES) index reported for each pathway as median (IQR; 95% CI), arranged from top to bottom. Significant differences, based on a significance level of .05, are highlighted in green. Pie charts indicate the relative frequencies of participants at each stage. Paths with fewer than 5 participants are excluded from the analysis. LAR: Legally Authorized Representative.

While no significant differences in the HOUSES index were observed across different participation pathways, participants who did not respond to the initial invitation had significantly higher ADI national ranks (median 45.0, IQR 29.0-63.0; 95% CI 44.0‐46.0) compared to those who expressed interest (median 42.0, IQR 27.0-59.0; 95% CI 39.0‐43.0), indicating residence in more socioeconomically disadvantaged neighborhoods. The narrow CIs for these ADI estimates suggest precise measurement of this disparity, underscoring the influence of neighborhood-level socioeconomic disadvantage on initial engagement. No other significant differences in ADI national ranks were observed across subsequent recruitment steps. Figure 3 details the participant flow and checkpoint-specific distributions.

## Discussion

### Summary of Principal Findings

This study provides a comprehensive analysis of participant recruitment pathways in a digital speech research study, revealing important associations between sociodemographic factors and participation outcomes. Understanding these pathways is critical for improving equity and efficiency in digital clinical research [17]. First, recruitment remains a fundamental challenge in clinical research, with participation rates often below 10 percent in specialized studies, limiting generalizability and slowing innovation [1,2]. Second, as digital recruitment methods become increasingly prevalent, it is essential to assess whether these approaches truly reduce barriers or inadvertently perpetuate existing disparities, an area where evidence remains limited [8]. By analyzing longitudinal recruitment data from 5846 invited patients, our findings demonstrate that systematic pathway analysis can uncover patterns of participation and attrition that may not be apparent when examining only final enrollment outcomes. These results support our primary hypothesis that sociodemographic factors, including age, urbanicity, and neighborhood disadvantage, are associated with recruitment and attrition in digital research workflows. This finding challenges the assumption that digital methods inherently improve inclusivity and highlights the need for targeted strategies, such as age-specific engagement and rural digital support, to promote retention in remote research.

### Age-Related Participation Patterns

The significant age differences observed at various dropout points suggest that digital recruitment methods may be less effective for younger populations. Our analysis showed that the median age increased from 63 (IQR 48-72; 95% CI 62‐63) years in the invited cohort to 66.4 (IQR 56.0-72.5; 95% CI 65.1‐67.6) years among completers, indicating a robust and precise trend toward older participant retention. This finding challenges the conventional wisdom that digital methods inherently appeal to younger participants and suggests that age-specific engagement strategies may be necessary throughout the recruitment process. While younger individuals may be more comfortable with technology, our results and others suggest that younger participants are more likely to disengage or drop out of digital studies over time [27,28]. Possible explanations include competing priorities, lower perceived relevance of neurological research, and reduced tolerance for multistep enrollment processes [29]. This emphasizes the importance of age-sensitive retention strategies that extend beyond initial recruitment, including tailored messaging and incentives to support long-term engagement.

### Disparities by Urbanicity in Enrollment Timing

The observation that participants from urban areas completed enrollment significantly faster than those from rural areas or urban clusters highlights potential disparities by urbanicity in research accessibility. Urban participants enrolled in a median of 32.0 (IQR 9.0-58.0; 95% CI 31.0‐37.0) days compared to 41.0 (IQR 22.0-65.0; 95% CI 37.0‐49.0) days for rural and 40.0 (IQR 13.0-71.0; 95% CI 33.0‐49.0) days for urban clusters, underscoring a consistent and precise difference. This finding aligns with broader concerns about the urban-rural digital divide and suggests that digital recruitment, while theoretically boundaryless, may still be influenced by infrastructure, digital literacy, or health care engagement [30,31]. Rural participants may face slower internet speeds, limited device availability, and less familiarity with patient portals, which could delay enrollment [32]. This contrasts with some findings in telehealth adoption, which suggest that technology can overcome location-based care barriers [33]. To mitigate these disparities, future strategies should include technical support for rural participants, alternative enrollment options (eg, phone-based consent), and targeted outreach through local health care networks.

### Socioeconomic Disadvantage and Participation

Perhaps most notably, our analysis revealed that participants from neighborhoods with higher socioeconomic disadvantage (higher ADI national ranks) were significantly less likely to respond to initial invitations. This may reflect limited broadband access, lower digital literacy, and competing priorities in disadvantaged neighborhoods [34]. This finding is consistent with prior research demonstrating that individuals from more disadvantaged areas experience greater barriers to engaging with digital health, including higher no-show rates and lower uptake of telehealth services [35,36]. Our study adds nuance by comparing neighborhood-level (ADI) and housing-based (HOUSES) SES measures, an approach rarely examined in digital recruitment literature. This finding suggests that digital recruitment methods may perpetuate existing socioeconomic disparities in research participation if not specifically designed to address these barriers. To mitigate these barriers, recruitment strategies should include targeted outreach in areas with high ADI, provide technical support, and incorporate offline options. The absence of significant differences in the HOUSES index across participation pathways, despite differences in the ADI national rank, suggests that neighborhood context likely influences digital engagement more than individual housing characteristics because infrastructure and community resources shape access and literacy.

### Sex Differences in Enrollment Dynamics

Females took longer to complete the enrollment process than males, a difference that warrants further investigation. This may reflect differences in time availability, competing responsibilities, or engagement with digital health platforms that could impact recruitment strategies. Prior studies suggest that social and structural factors, such as unequal distribution of caregiving and household responsibilities, affect time availability for research participation, particularly in remote studies [37]. Recruitment workflows could incorporate flexible scheduling and simplified enrollment steps to reduce time burden. While underexplored in the current literature, these insights underscore the need for sex- and gender-aware design in digital recruitment, which may improve inclusivity and reduce attrition.

### Recruitment Funnel Attrition

Of 5846 individuals invited, a large proportion did not respond or declined participation, and only 415 of all the invited individuals (7.1%) completed the study. This significant attrition, consistent with patterns observed in other digital recruitment efforts [38,39], may reflect perceived complexity of enrollment, lack of immediate incentives, and competing priorities among participants. These findings highlight the need for iterative, multitouch recruitment strategies that re-engage potential participants and address barriers to active enrollment. Such strategies could include reminder messages, simplified consent processes, and personalized follow-ups to maintain engagement. Our findings reinforce prior evidence that digital recruitment alone is insufficient and highlight the importance of hybrid approaches combining digital and traditional outreach strategies [40].

### Limitations

Several limitations should be considered when interpreting these findings. First, this was an observational analysis conducted within a single academic health system, which may limit generalizability to other settings with different patient demographics or digital infrastructure. Second, the study relied on electronic health record–based digital recruitment and patient portal messaging, which presumes access to broadband internet and digital literacy; these factors were not directly measured and may have influenced participation patterns. Findings may generalize to similar academic health systems using portal-based recruitment but may differ in settings with lower portal adoption or different sociodemographic profiles. Third, the cohort was predominantly White (93.7%), limiting the ability to examine racial and ethnic disparities in digital recruitment. Future studies should prioritize inclusion of more diverse populations to assess whether similar sociodemographic patterns persist across racial and ethnic groups. Fourth, while we examined neighborhood-level (ADI) and housing-based (HOUSES) socioeconomic measures, other dimensions of socioeconomic status, such as income, education, and employment, were not available and could provide additional insights. Fifth, attrition analysis was based on recruitment checkpoints rather than qualitative data on participant motivations or barriers, which constrains the interpretation of underlying causes for dropout. Finally, the study period was relatively short (March to July 2024), and findings may not reflect seasonal or long-term trends in digital recruitment dynamics.

To address these challenges, we recommend several strategies to enhance inclusivity and effectiveness in digital recruitment. First, adopting a multichannel approach is essential. Digital methods should be complemented with traditional outreach, particularly for populations with limited online access, such as those in rural areas or socioeconomically disadvantaged communities. Second, age-specific engagement should be prioritized by developing tailored messaging and user experiences for different age groups, with special attention to reducing dropout rates among younger participants. Similarly, geographic barriers must be addressed by providing technical support and offering alternative participation options for both rural and urban clusters. To ensure continuous improvement, it is critical to track recruitment analytics. Monitoring data will help identify dropout points and demographic trends, enabling real-time adjustments to recruitment strategies. Additionally, efforts should be made to minimize participation burden by streamlining enrollment processes to reduce time demands, which is particularly beneficial for individuals with limited availability, such as those with caregiving responsibilities. Socioeconomic factors also require consideration. Recruitment materials should be inclusive, and incentives or support should be offered to offset participation costs, thereby improving socioeconomic accessibility. Furthermore, while device type did not affect completion time in our findings, platforms should still be optimized for device diversity, ensuring mobile-friendly and cross-device accessibility. Ultimately, intentional design and continuous evaluation are key to ensuring that digital methods promote inclusivity rather than hinder it. Future work should explore the mechanisms behind demographic and socioeconomic disparities, test interventions to address these patterns, and evaluate whether similar dynamics occur across other clinical domains.

### Conclusion

This study demonstrates that digital recruitment methods in neurological research are subject to demographic, urbanicity, and socioeconomic influences that affect the representativeness of study populations. By mapping these factors, this study provides actionable insights for designing recruitment strategies that improve participation in remote neurological research and similar digital health initiatives. Our findings suggest that although digital recruitment expands reach, it does not eliminate traditional barriers and introduces new challenges. The digital divide appears to manifest in nuanced ways throughout the recruitment process, potentially influencing who participates in neurological research and, consequently, who benefits from its findings. The relatively low overall completion rate (7.1% of invited participants) underscores the persistent challenge of recruitment in specialized medical research, even with digital methods. Collectively, these findings reinforce the importance of refining digital recruitment strategies to bridge the persistent digital divide and promote research participation.

## Supplementary material

10.2196/83432 Multimedia Appendix 1 American Psychological Association JARS-Quant guidelines, completed checklist.

## Abbreviations

ADI: Area Deprivation Index
AI: artificial intelligence
APA: American Psychological Association
HOUSES: Housing-based Socioeconomic Status
JARS: Journal Article Reporting Standards
MCAR: missing completely at random
PTrax: Participant Tracking System
SES: socioeconomic status
