# Supplementary material for: Sociodemographic Drivers of Recruitment and Attrition in Digital Neurological Research: Longitudinal Cohort Study
Source: J Med Internet Res. 2026 Feb 25;28:e83432. doi: 10.2196/83432 (PMC12935417; doi:10.2196/83432)
Supplement: Multimedia Appendix 1 [file jmir-v28-e83432-s001.pdf]

JARS–Quant | Table 1: Manuscripts Reporting New Data Collections

Title and Title Page

Title: Sociodemographic Drivers of Recruitment and Attrition in Digital Neurological Research: Longitudinal Cohort Study

Author Note:

- Registration: Not registered (observational secondary analysis).
- Prior use of data: Not previously published, secondary analysis of operational data from an ongoing speech-capture study.
- Funding/support: UL1TR002377 (NCATS), R01AG083832 (NIA).
- Conflicts of interest: Authors declared none.
- Corresponding author: Hugo Botha; 200 First St. SW, Rochester, MN 55905; Botha.Hugo@mayo.edu.
- Affiliations: Departments and offices at Mayo Clinic (Anesthesiology & Perioperative Medicine; Office of Digital Innovation; Neurology; Information Technologies).
- Generative AI disclosure: Use of GPT-5 within Microsoft Copilot for literature search/systematization, code generation/optimization, proofreading/editing, summarizing, reformatting (under full human supervision).
- Data availability: De-identified datasets available upon reasonable request with institutional approvals.

Abstract

Objectives: Investigate how sociodemographic factors influence recruitment and attrition in a remote neurological research cohort, map participation pathways, and identify disparities to inform inclusive digital strategies.

Participants: 5,846 invited adult patients (≥18 years, U.S. residents, English-speaking) identified from Mayo Clinic EHR (Epic), demographics summarized (age median 63 years; 56.2% female; 93.7% White).

Study Method: Nonexperimental, observational longitudinal cohort design using patient-portal invitations (March–July 2024). Data sources: Epic (demographics), Qualtrics (screening), PTrax (e-consent), speech recording platform (completion). Measures: ADI national rank; HOUSES index; age; sex; urbanicity (urban, rural, urban cluster).

Findings: 415/5,846 (7.1%) completed all requirements. Completers were older (median 66.4 vs 62.8 years; $P<.001$). Nonresponders had higher ADI ranks (median 45 vs 42; $P<.001$). Urban

participants enrolled faster than rural/urban cluster (median 32 vs 41/40 days; P=.01). Females enrolled slower than males (median 38.5 vs 32 days; P=.01). HOUSES and device type not associated with completion/timelines. Missingness MCAR (χ^2(df=3)=3.45; P=.24).

Conclusions: Digital recruitment does not automatically mitigate traditional barriers and may introduce disparities by age, urbanicity, and neighborhood disadvantage, recommend multi-channel outreach, age-specific engagement, and rural technical support.

Introduction

Problem: Recruitment in specialized clinical research is challenging, digital methods show inconsistent effects on diversity and efficiency and may reflect the digital divide.

Review of Relevant Scholarship: Summarizes evidence across digital recruitment tools (EHR portals, social media, apps), digital divide impacts, and mixed-channel strategies. Cites recent systematic reviews and domain-specific studies.

Hypotheses/Aims/Objectives: Primary objective to assess associations between sociodemographic factors (age, sex, urbanicity, ADI, HOUSES) and participation outcomes (completion, time to enrollment). Secondary descriptive aims include mapping pathway-specific dropout and enrollment timelines and exploratory analyses of device type. No formal primary/secondary hypothesis hierarchy formed, analyses are observational and descriptive.

Method

Inclusion and Exclusion: Inclusion: adults ≥18 years; U.S. residence; English communication ability. Exclusion: outside U.S.; non-English; under 18.

Participant Characteristics: Invited cohort N=5,846; age 18–96 (median 63, IQR 48–72); sex: 56.2% female, 43.8% male, 0.05% unknown; race: 93.7% White, 1.7% Black/African American, 1.1% chose not to disclose, 3.5% other; urbanicity: 56.5% urban, 23.3% rural, 20.2% urban cluster.

Sampling approach: Convenience sampling via patient portal invitations to patients with upcoming neurology appointments at Mayo Clinic (Rochester, MN). Self-selection occurred based on response to invitations and eligibility survey.

Setting and dates: Data collected March–July 2024.

Agreements and payments: No compensation was provided.

Ethical oversight: Mayo Clinic IRB approval for the overarching speech capture study (IRB #22-002430). Secondary analysis used de-identified data; no additional IRB required.

Intended sample size: Not predetermined for this observational analysis (uses all eligible patients invited during the window). Achieved sample size: 5,846 invited; 1,358 expressed interest; 415 complete participants.

Determination of sample size: No a priori power calculation; precision conveyed using 95% confidence intervals for medians and IQRs.

Interim analyses/stopping rules: None used.

Measures and Covariates

- Primary outcomes: Study completion; time to enrollment and completion.
- Predictors/exposures: Age; sex (assigned at birth); urbanicity category; ADI national rank; HOUSES index percentile.
- Potential confounders: Device type; urbanicity (in analyses of SES indices).
- Measures collected but not reported: None beyond those listed.

Data Collection: Epic (demographics), Qualtrics (eligibility responses), PTrax (consent tracking), patient portal messages, and the speech recording platform (task completion). Longitudinal timestamps recorded at each checkpoint.

Quality of Measurements: Standard operational systems generated all statuses and timestamps; data were de-identified before analysis. No specialized training of data collectors or additional measurement procedures were implemented for this secondary analysis.

Instrumentation: Institutional systems: Qualtrics XM (eligibility), AdobeSign via PTrax (e-consent), recording platform for speech tasks. Psychometric properties not applicable (no scales requiring reliability/validity).

Masking: Not applicable; no experimental manipulation or condition assignments. Observational tracking only.

Psychometrics: Not applicable; study relies on administrative/operational data and socioeconomic indices with established external validity (ADI, HOUSES). Reliability coefficients were not estimated on this sample.

Conditions and Design: Nonexperimental (observational) longitudinal cohort design; naturally observed conditions. JARS–Quant Tables relevant: Table 3 (nonexperimental designs) and Table 4 (longitudinal designs).

Data Diagnostics

- Exclusion criteria post-collection: Paths with <5 participants without missing values were excluded from statistical comparisons to ensure validity.
- Missing data: Little's MCAR test $\chi^2(df=3)=3.45$, P=.24; pairwise deletion applied.

- Outliers and transformations: No outlier removal; no variable transformations; Anderson–Darling tests indicated non-normality for age, ADI, HOUSES ($P<.001$).

Analytic Strategy: Nonparametric tests (Wilcoxon rank-sum, Kruskal–Wallis) for group comparisons of medians and timelines; exact P-values reported with 95% Cis for medians. **Software**: BlueSky Statistics v10.3.4; Python SciPy v1.16. No formal correction for multiple testing reported.

Results

Participant Flow: Invited: 5,846; No response to invitation: 2,736; Not interested: 1,752; Expressed interest: 1,358; Completed all requirements: 415 (7.1%). 95 withdrew consent post-consent prior to accrual. Total nonresponses to follow-up across stages: 3,346. Figures illustrate flow and checkpoint-specific distributions.

Recruitment: Recruitment and data collection period: March–July 2024. Enrollment/completion timelines analyzed across urbanicity and sex.

Statistics and Data Analysis

- Missing data frequencies: ADI available for 5,403/5,846 (7.6% missing); HOUSES available for 5,439/5,846 (7.0% missing); age available for all invited; sex unknown for 3/5,846 (0.0% missing).
- Missingness mechanism: MCAR supported by Little's test ($\chi^2(df=3)=3.45$, $P=.24$).
- Primary outcomes and inferential results: Completers older than non-completers (median 66.4 vs 62.8 years; $P<.001$). Completers had slightly lower ADI ranks than non-completers (median 41.0 vs 44.5; $P=.04$). Enrollment time faster for urban vs rural/urban cluster (median 32 vs 41/40 days; $P=.01$). Females enrolled slower than males (median 38.5 vs 32.0 days; $P=.01$).
- Effect-size estimates: Not calculated; manuscript reports exact P-values and 95% CIs for medians.
- Software and estimation: BlueSky Statistics v10.3.4 and Python SciPy v1.16 used; nonparametric tests due to non-normal distributions.

Discussion

Support of original aims/hypotheses: Findings support the premise that age, urbanicity, and neighborhood disadvantage are associated with recruitment and attrition in digital research.

Similarity of results: Patterns align with literature on digital divide and portal-based recruitment; mixed-channel strategies are recommended in prior work.

Interpretation: Observational single-system context; potential biases include portal access literacy; predominantly White cohort limits race/ethnicity analyses; no effect sizes; multiple comparisons not adjusted.

Generalizability: May generalize to similar academic health systems using portal-based recruitment; context (setting, time frame, measurement via operational systems) should be considered.

Implications: Implement multi-channel outreach; age-specific engagement; rural technical support; track recruitment analytics; streamline enrollment to reduce burden; optimize platform for device diversity.

JARS–Quant | Table 3 and 4: Nonexperimental Designs, and Longitudinal Studies

Data Use

Type of data: Secondary analysis of operational data from an ongoing speech-capture study; sources include Epic EHR, Qualtrics, PTrax, and recording platform.

Participant Selection

Selection method: Convenience sampling via patient portal invitations to Mayo Clinic neurology patients with upcoming appointments (March–July 2024).

Groups: Natural groups formed by participation status (completers vs non-completers).

Matching: None applied.

Data sources: Epic for demographics; Qualtrics for eligibility; PTrax for consent; recording platform for completion.

Variables

Exposures/predictors: Age, sex, urbanicity, ADI national rank, HOUSES index percentile.

Outcomes: Study completion; time to enrollment and completion.

Measurement: Age from EHR; sex from EHR; ADI and HOUSES from geocoded addresses; urbanicity from census classification.

Comparability of Assessment

All groups assessed using identical operational systems; likelihood of observing outcomes consistent across groups (portal-based tracking).

Analysis

Predictors and confounders included in nonparametric tests (Wilcoxon, Kruskal-Wallis). Device type considered as exploratory covariate. No propensity matching or multivariable modeling performed.

Limitations

Potential limitations: Self-selection bias; predominantly White cohort limits race/ethnicity analysis; possible misclassification of SES indices; unmeasured confounders (digital literacy); no effect sizes reported; multiple comparisons without correction.

Sample Characteristics

Unit: Individual patients. N=5,846 invited; age median 63 (IQR 48–72); sex: 56.2% female; race: 93.7% White; SES indices (ADI, HOUSES) reported; urbanicity distribution: 56.5% urban, 23.3% rural, 20.2% urban cluster.

Sample Recruitment and Retention Methods

Recruitment: Patient portal invitations; retention tracked through eligibility, consent, and task completion checkpoints.

Attrition

Attrition by stage: Invited 5,846 → no response 2,736 → not interested 1,752 → interested 1,358 → completed 415 (7.1%). 95 withdrew post-consent.

Differential attrition: Older participants more likely to complete; urban participants enrolled faster.

Additional Sample Description

Contextual changes: None reported during study period (March–July 2024).

Method and Measurement

Independent variables: Age, sex, ADI, HOUSES, urbanicity. Dependent variables: Completion status, time to enrollment/completion. Waves: Invitation, eligibility, consent, recording.

Missing Data

ADI missing for 7.6%; HOUSES missing for 7.0%; sex missing for 0.05%. Little's MCAR test $\chi^2(df=3)=3.45$, $P=.24$; pairwise deletion applied.

Analysis

Approach: Nonparametric tests for group comparisons; assumptions: non-normal distributions confirmed by Anderson-Darling tests; software: BlueSky Statistics v10.3.4 and Python SciPy v1.16.

Multiple Publication

Data not previously published; secondary analysis of operational data from an ongoing study.
